# Nanometre-scale 3D defects in Cr_2_AlC thin films

**DOI:** 10.1038/s41598-017-01196-3

**Published:** 2017-04-20

**Authors:** Y. T. Chen, D. Music, L. Shang, J. Mayer, J. M. Schneider

**Affiliations:** 1 Materials Chemistry, RWTH Aachen University, Kopernikusstr. 10, 52074 Aachen, Germany; 2 Central Facility for Electron Microscopy, RWTH Aachen University, 52056 Aachen, Germany; 3 Ernst Ruska-Centre for Microscopy and Spectroscopy with Electrons, Forschungszentrum Juelich, 52425 Juelich, Germany

## Abstract

MAX-phase Cr_2_AlC containing thin films were synthesized by magnetron sputtering in an industrial system. Nanometre-scale 3D defects are observed near the boundary between regions of Cr_2_AlC and of the disordered solid solution (CrAl)_x_C_y_. Shrinkage of the Cr-Cr interplanar distance and elongation of the Cr-Al distance in the vicinity of the defects are detected using transmission electron microscopy. The here observed deformation surrounding the defects was described using density functional theory by comparing the DOS of bulk Cr_2_AlC with the DOS of a strained and unstrained Cr_2_AlC(0001) surface. From the partial density of states analysis, it can be learned that Cr-C bonds are stronger than Cr-Al bonds in bulk Cr_2_AlC. Upon Cr_2_AlC(0001) surface formation, both bonds are weakened. While the Cr-C bonds recover their bulk strength as Cr_2_AlC(0001) is strained, the Cr-Al bonds experience only a partial recovery, still being weaker than their bulk counterparts. Hence, the strain induced bond strengthening in Cr_2_AlC(0001) is larger for Cr d – C p bonds than for Cr d – Al p bonds. The here observed changes in bonding due to the formation of a strained surface are consistent with the experimentally observed elongation of the Cr-Al distance in the vicinity of nm-scale 3D defects in Cr_2_AlC thin films.

## Introduction

M_n+1_AX_n_ phases (henceforth MAX, where M: transition metals, A: IIIA or IVA elements, X: C or N, n = 1 to 3) have attracted a lot of attention^1–3^ due to their outstanding mechanical properties^4^, good corrosion resistance^5^, good thermal stability^6, 7^, and large electrical and thermal conductivity^1^. Cr_2_AlC has been reported to exhibit self-healing behaviour^8, 9^. A large number of theoretical predictions on MAX phase materials has been published, being focused on the calculation of energetics^10, 11^, electronic structure^10^, thermal properties^12, 13^, growth phenomena^2^, tribological^2^, mechanical^12–14^, and magnetic properties^15^. Furthermore, defects in MAX phases have been studied, including point-defects induced by intrinsic impurities^16^, which imply antisite and interstitial incorporation^8, 9^, as well as extrinsic impurities, such as O, which can be relevant for self-healing applications^17^. Emmerlich *et al*.^6^ reported the merge of voids with tens of nanometers in size and phase transformations occurring during the decomposition of Ti_3_SiC_2_(0001). The same group observed the formation of voids on the scale of tens of nanometers during the epitaxial growth of Ti_3_SiC_2_ in the vicinity of the substrate or when tilted basal planes grow into each other^18^. Also, Eklund *et al*.^19^ reported the formation of pores with tens of nanometres in the homoepitaxial growth of Ti_3_SiC_2_ thin film on Ti_3_SiC_2_ bulk. In all of the above cited papers, the presence of 3D defects on the scales of several to hundreds of nanometres, has been reported, but not studied with atomic resolution by theoretical or experimental methods. Furthermore, reports on the lattice distortions and corresponding electronic structure changes in the vicinity of these defects are not available.

In this study, STEM-HAADF (scanning transmission electron microscopy with high angle annular dark field) investigations are performed focusing on the analysis of interplanar distances in the vicinity of a 3D defect. Cr_2_AlC, compared to the commonly discussed Ti_2_AlC and V_2_AlC, has a higher bulk modulus of 36% and 17%, respectively^20^, and superior performance for self-healing applications^21^. Therefore, it is chosen as a model MAX phase system in this investigation. Ab initio calculations are conducted to rationalize the observed deformation in the vicinity of these defects. As defects in general may act as nucleation sites for oxide formation during oxidation at elevated temperatures, the fact that these previously-overlooked 3D defects occur in MAX phases is of fundamental importance for self-healing behaviour of MAX phases.

## Result and Discussion

**Figure 1:**
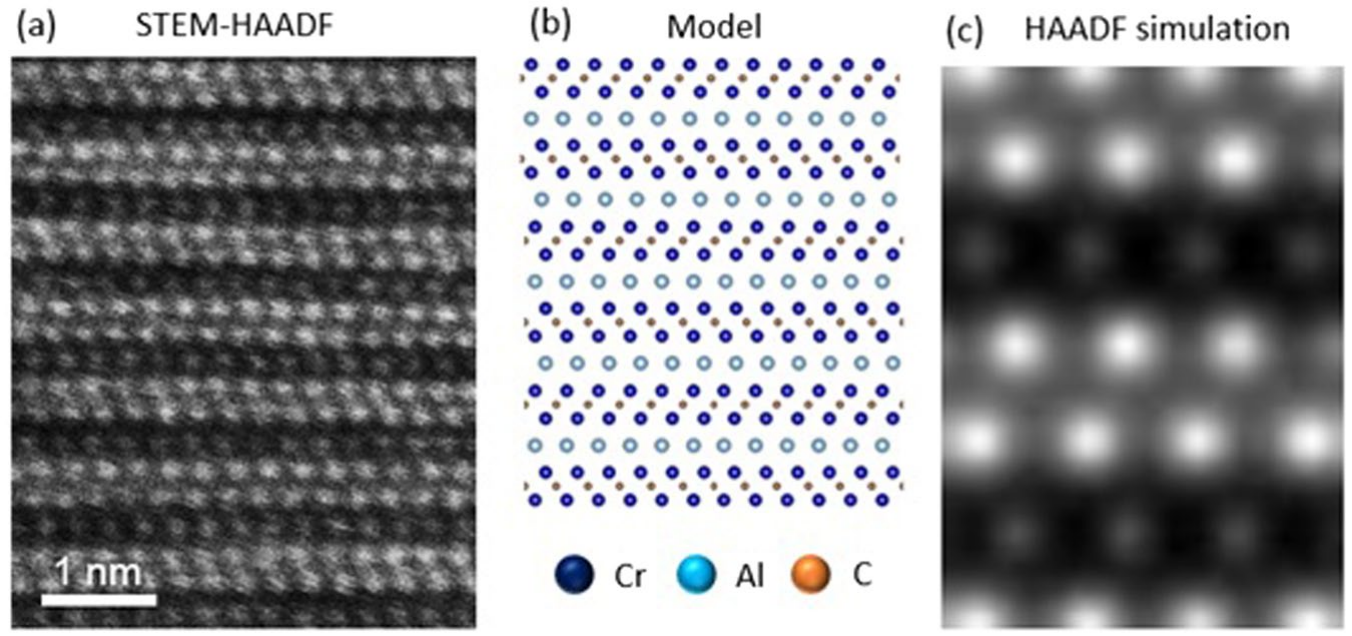
(**a**) Unprocessed STEM-HAADF image in the direction of [1010] shows the Z-contrast distribution of chromium and aluminium atomic columns in the MAX phase structure. (**b**) A model of Cr_2_AlC MAX phase structure, (**c**) Simulation result of STEM-HAADF under consideration of partial temporal and spatial coherence. The probe is assumed to have no aberration.

A STEM-HAADF was performed with the crystal orientation [1010] out of the plane of the figure, as shown in Fig. 1(a). It was carried out with a FEI 80–300 Titan field emission electron microscope^22^ equipped with a spherical aberration corrector element (CEOS) in the probe-forming illumination system along with an electron monochromator and a post column energy filter system (Gatan), operating at 300 kV. Due to the nature of the Z-contrast, chromium atoms exhibit a much higher intensity than the aluminium atoms in the image, while the carbon atoms are invisible owing to their small contrast in the HAADF image. Hence, Cr- and Al-containing atomic columns are clearly visible. No evidence for the presence of dislocations was obtained. For the calculation, a Cr_2_AlC supercell with periodic boundary conditions was constructed. A relaxation process was performed beforehand to obtain the equilibrium lattice constants, as shown in Fig. 1(b). In addition, a simulation of the STEM-HAADF image was carried out using the Cr_2_AlC supercell with the multi-slice method, as shown in Fig. 1(c). An in-house developed software (Dr. Probe)^23^ was used for the STEM image simulation. In the simulation, partial spatial coherence was considered by subsequent convolution of the effective source profile, which was set with a Lorentz distribution of 0.05 nm as the value of half width half maximum. Partial temporal coherence was considered by explicit focal averaging^23^. The probe was set to zero aberration. The slice boundaries were defined to avoid cutting through atomic sites. The acceleration voltage was set to 300 kV, and a convergence angle of 25 mrad was used in order to match the experiments. The inner and outer radii of HAADF detector were set to 80 mrad and 250 mrad, respectively. The experimental intensity of Cr and Al in the TEM image shown in Fig. 1(a) are 216 and 64 (arbitrary unit), respectively, having a ratio of 3.375. The calculated intensity in the STEM simulation shown in Fig. 1(c) are 232 and 63, exhibiting a ratio of 3.682, which deviates 8.4% from the measurement. Based on the model/observation/simulation in Fig. 1, we delineate the MAX phase surface inside the pore and provide a rationale for the electronic structure description, as shown below. The STEM image simulation shows only the ideal MAX phase structure. To describe the structure in the vicinity of the pore, several thousand atoms are required, which is not feasible with the here employed state-of-the-art ab initio code.

**Figure 2:**
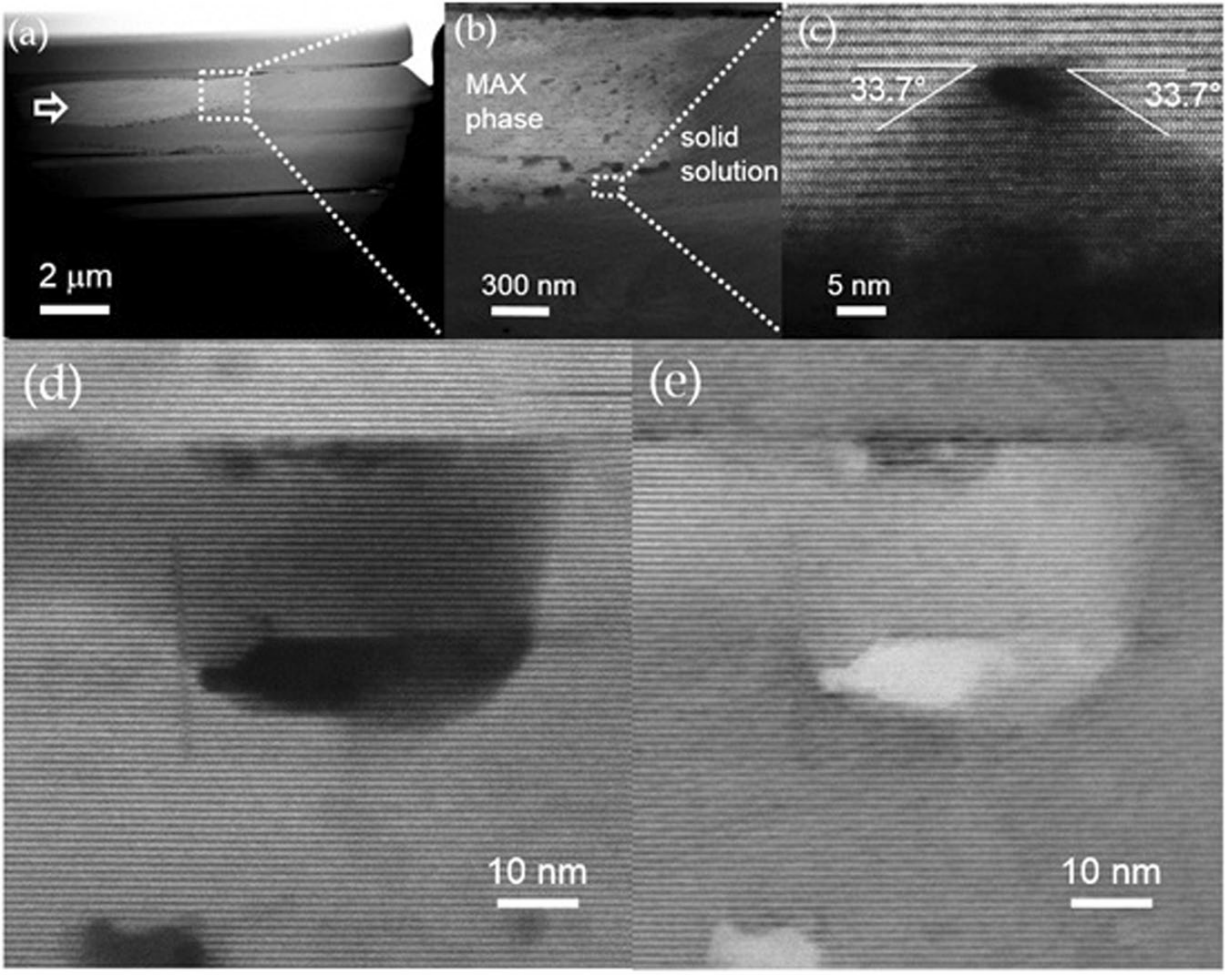
STEM-HAADF investigation. (**a**) Image of the Cr_2_AlC lamella exhibiting a columnar morphology. For all images in Fig. 2, the growth direction is from the left to the right. The dashed square indicates the region enlarged in (**b**). (**b**) Enlarged image of the location near the boundary of MAX phase area (brighter left part) with an increased defect density. (**c**) One defect is depicted as an example. Defects populate the boundary region between MAX phase and disordered solid solution. Simultaneously recorded dark-field and bright-field STEM images are shown in (**d**) and (**e**), respectively. The defects shown as black area in dark-field images appear to be white in the bright-field image. No sign of lattice fringes can be found in the defect, as a signature of filling with light elements or vacuum.

A STEM image of a Cr_2_AlC sample is shown with a low magnification in Fig. 2(a). The sample exhibits a columnar morphology. A Cr_2_AlC MAX-phase column (area identified by an arrow in Fig. 2(a)) exhibits a higher intensity due to the zone axis orientation of the crystal with respect to the electron beam. 3D defects can be found at the boundary of the Cr_2_AlC MAX phase – disordered (CrAl)_x_C_y_ solid solution regions, as shown in Fig. 2(b). The density of defects increases along the growth direction (left to right in the image). For some defects, the crystalline orientations of the boundaries are aligned with (0118) and (0118) planes symmetrical to each other, and with the angles of 33.7° to the basal plane, as shown in Fig. 2(c) here. The possibility of FIB induced nanometre scale 3D-defect formation has been considered as well. However, owing to the fact that the defects only appear at the boundary of MAX phase and solid solution regions shown in Fig. 2(a) as well as that some of the defects appear to exhibit a structural relationship with the film orientation, as shown in Fig. 2(c), it is evident that these defects are related to the crystal growth by vapour phase condensation and are hence not an artefact of the sample preparation. Also, considering the base pressure of 4 × 10^−6^ Torr and our previous work on residual gas incorporation during magnetron sputtering^24^ as well as on oxygen incorporation in Cr_2_AlC^8^, we expect an oxygen concentration on the at.% level. However, as the 3D-defects appear to exhibit a structural relationship with the film orientation, it is unlikely that their formation is caused by the incorporation of oxygen.

Further analysis of the 3D defects is conducted using dark field and bright field images, as shown in Fig. 2(d) and (e). The high intensity observed in the HAADF image depicting the defect in Fig. 2(d) is consistent either with the presence of light elements such as carbon, or with the absence of material (vacuum). As no crystalline fringes can be discerned in Fig. 2(d) and (e) it is reasonable to assume that either vacuum or an amorphous, light element phase fills the defect. For the theoretical discussion below we assume that the defect represents a nm scale void.

**Figure 3:**
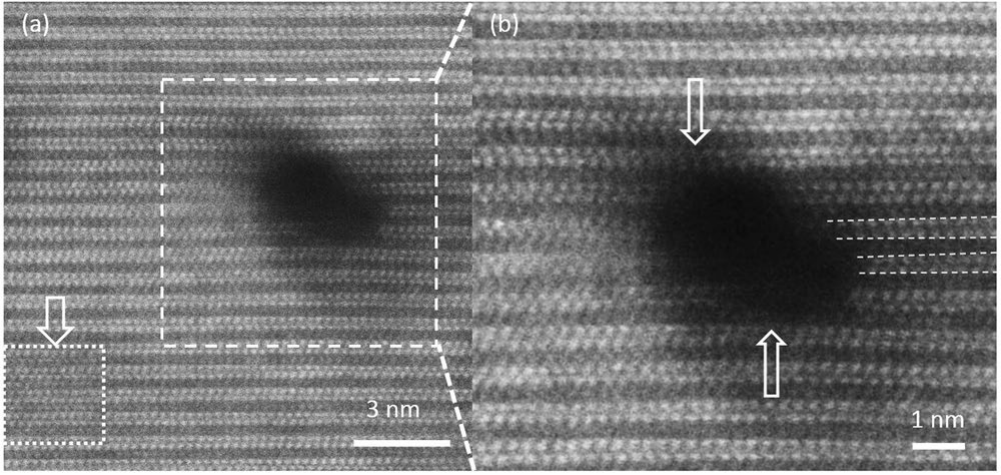
(**a**) Higher magnification image clearly showing the lattice distortion around the defect, where all the planes defined by Cr atoms tend to bend towards the defect. The area with structure changed by the electron beam is indicated by a rectangle. (**b**) Enlargement of the defect shown in (**a**). The lattice distortion can be observed at the positions along the direction of the upper and lower arrows. In addition, the Cr-Cr interplanar distance is shortened towards the defect, while the Cr-Al distances are elongated, as indicated with dashed lines.

High magnification images of a 3D defect are shown in Fig. 3(a) and (b). A lattice distortion in its vicinity is clearly visible while at a distance of approximately 5 nm from the defect the lattice spacing appears unstrained. Based on the image in Fig. 3(b) the change in interplanar distances can be estimated to be on the order of 10% to 20%, bending along the direction of the basal-plane towards the defect as indicated by the arrows. Interestingly, Cr-Cr interplanar distances are shortened, while the Cr-Al distances are increased compared to the position of atoms in the strain-free region of the specimen. Based on the difference in thermal expansion coefficient between Cr_2_AlC^25^ and Al_2_O_3_^26^ the as deposited thin film is expected to exhibit a tensile stress state after cooling down from the deposition temperature. However, Ar ion bombardment^27^ as well as bombardment with ions of the film forming species^28^ are expected during thin film growth by HPPMS. It is well known that film grown in the presence of ion bombardment can exhibit compressive stress states^29^ consistent with the lattice deformation around the defect observed in the STEM images shown in Fig. 3(a) and (b). More images of lattice distortion in the vicinity of defects are shown in Figure S1 of supplementary information. To exclude the artefacts of knock on damage from the 300 kV electron beam, we have focused the electron beam on a small region and observed the change of the atomic contrasts under the electron beam illumination, as indicated by a rectangle in Fig. 3(a). Since the defect population is high and is gradually increased from the MAX phase region to the solid solution region (Fig. 2a and b) and since some of the defects are located at the intersection of two symmetric crystal planes (Fig. 2c), it is reasonable to assume that the defects are not created by the electron beam. The probability that the electron beam is residing exactly on the intersection of symmetric crystal planes is extremely small indeed. Hence, these nano-meter scale 3D defects are formed during thin film deposition.

**Figure 4:**
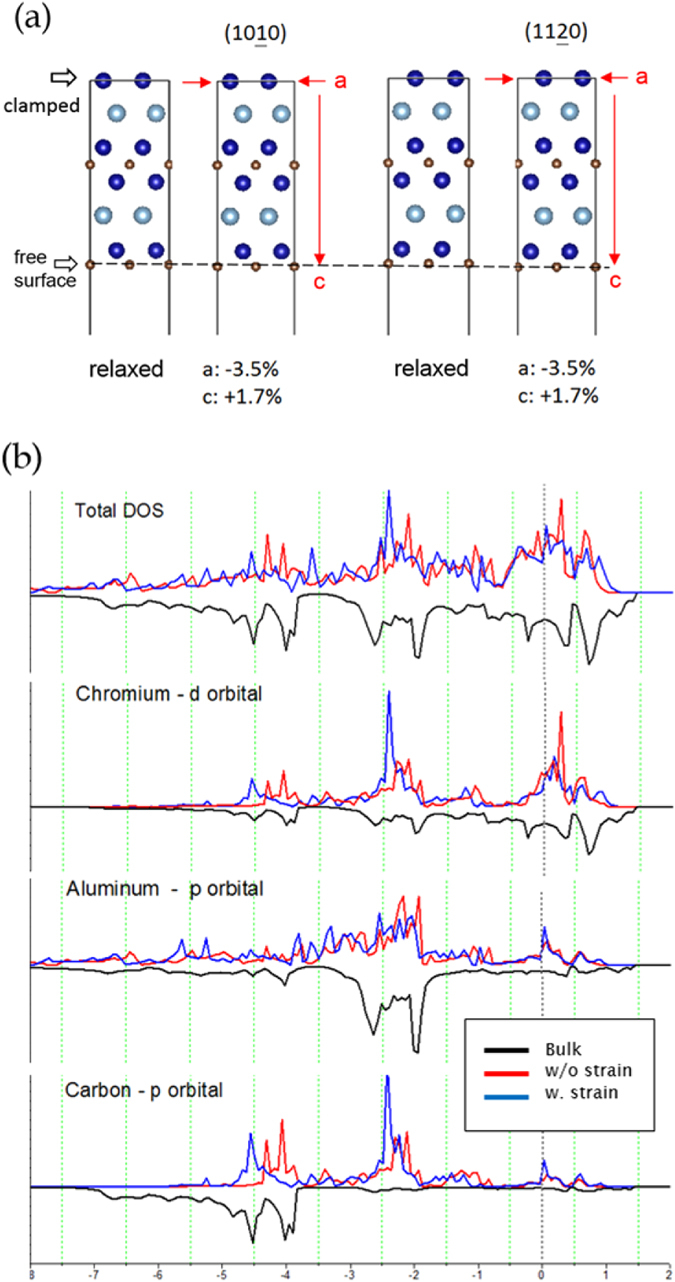
Density functional theory investigations. Structured model for Cr_2_AlC with periodic boundary conditions in the lateral directions with applied strain (**a**). Calculated density of states (DOS in arb. units) for Cr_2_AlC (**b**): total, partial chromium d states, aluminium p states, and carbon p states. The Fermi level is set to 0 eV. The comparison of the DOS data is focused on the atoms from the bulk, atoms close to the surface without strain, and atoms close to the surface with strain applied.

In order to describe the electronic structure in the vicinity of a 3D defect we assume that defect represents a nm scale void. Ab initio calculations were performed using the Vienna ab initio simulation package (VASP) within density functional theory^30^ applying a basis set in the form of projector augmented wave^31^ potentials, parameterized within the generalized-gradient approximation^32^. The Brillouin zone-integration was performed on Monkhorst-Pack k-points^33^ and the Blöchl corrections were applied for the total energy^34^. Supercells (2 × 2 × 1) containing 32 atoms were used on a mesh of 5 × 5 × 1 k-points. The convergence criterion for the total energy was 0.01 meV with a 500 eV cut-off. Three Cr_2_AlC configurations were explored: (i) bulk (periodic boundary conditions in all dimensions), (ii) a C-terminated surface without strain (periodic boundary conditions in the a-direction, vacuum layer in the z-direction, no strain), and (iii) a C-terminated surface with strain (periodic boundary conditions in the a-direction, vacuum layer in the z-direction, strain applied). For the basal surface model, as shown in Fig. 4(a), the slab was clamped in one end layer and free at the other side to describe the free defect surface. The lattice constants were changed in the a-direction to induce strain and subsequently the atomic positions were fully relaxed. When the crystal was exposed to the application of strain on a-direction caused an increase in lattice spacing in the z-direction.

Partial density of states (DOS) for Cr_2_AlC are shown in Fig. 4(b). It can be observed for the bulk Cr_2_AlC that the total DOS is continuous in the explored energy range and that a finite number of states are populated at the Fermi level. This implies extensive overlap of orbitals as well as metallic-like electrical and thermal conductivity, as commonly observed in MAX phases^2, 3^. Based on the partial DOS data, it is evident that the pronounced Cr d – C p, Cr d – Al p, and Cr d – Cr d orbital overlap occurs in the range of approximately −5.5 to −3.5 eV, −3.0 to −1.5 eV, and in the vicinity of the Fermi level (0 eV in the figure), respectively. This implies hybridization and hence covalent interaction between these constituents as well as that Cr-C bonds are stronger than Cr-Al bonds and that the metallic character is induced by Cr d states. Furthermore, the Fermi level is located at a distinct minimum between two sharp DOS peaks, indicating a strong overall bonding. This is consistent with literature^20, 35^. Moreover, this is also consistent with the reported bond energy data implying that Cr-C bonds are nearly threefold stronger than Cr-Al bonds^20, 36^.

Another configuration referred to as surface without (w/o) strain in Fig. 4(b) is considered. It is already evident from the total DOS data that the surface states fill the minimum observed in the electronic structure at the Fermi level of bulk Cr_2_AlC, which in turn implies lower stability and at least in part weaker bonds. This can further be analysed by partial DOS data while the hybridizations observed in the bulk Cr_2_AlC are still present, there are significant differences visible. Both Cr d – C p and Cr d – Al p hybridized states shift to higher energies, which suggests bond weakening, while the Cr d – Cr d states are less affected. These observations are consistent with literature^10, 11^.

To describe the electronic structure near the defect observed in our STEM analysis, we strain the Cr_2_AlC surface by 3.5% in the a-directions. The DOS data for this configuration, termed surface with strain, are also provided in Fig. 4(b). The hybridizations observed in the bulk Cr_2_AlC, which are initially shifted towards higher energies upon surface formation, are further affected by application of lateral strain. Both surface Cr d – C p and Cr d – Al p hybridized states shift to lower energies compared to the unstrained surface. However, the shift of the Cr d – C p states towards lower energies is larger than the shift of Cr d – Al p states, implying that the strain-induced bond strengthening is more pronounced for the Cr d – C p states recovering the bulk strength, while Cr d – Al p bonds remain weaker than those in the bulk configuration. Hence, the relative strength difference between the Cr-C and Cr-Al bonds when comparing bulk Cr_2_AlC and strained Cr_2_AlC(0001) becomes larger. Also in the strained state, the Cr d – Cr d states in the vicinity of the Fermi level, defining long-range interactions^10^ in this MAX phase, are less affected. This can be used to rationalize the presented experimental findings on interplanar distance modification in the vicinity of the defect. Based on the PDOS analysis it is predicted that defect formation which was simulated by the formation of a Cr_2_AlC(0001) surface causes bond weakening of Cr d – C p as well as Cr d – Al p bonds compared to the bulk. As the surface is strained, both the Cr d-C p and the Cr d – Al p bonds partially recover their bulk strength. However, the recovery is more pronounced for Cr d – C p bonds compared to the Cr d – Al p bonds. This is consistent with the observed shrinkage of Cr-C-Cr distance in the STEM-HAADF image and the elongation of Cr-Al interplanar distance observed in STEM, shown in Fig. 2(e). Based on our quantum mechanical data, the Cr-C bond in Cr_2_AlC(0001) exhibits the bond length of 1.872 Å. Upon straining, it shrinks to 1.852 Å by 1.1%, which may be interpreted as bond strengthening upon surface straining. Weaker Cr-Al bonds in Cr_2_AlC(0001) possess the bond length of 2.740 Å, which is elongated compared to the corresponding bulk bonds. As the (0001) surface is subsequently strained, it decreases to 2.719 Å by 0.7%, which is a smaller change (recovery) compared to stronger Cr-C bonds. These data are consistent with the DOS and TEM analyses.

**Figure 5:**
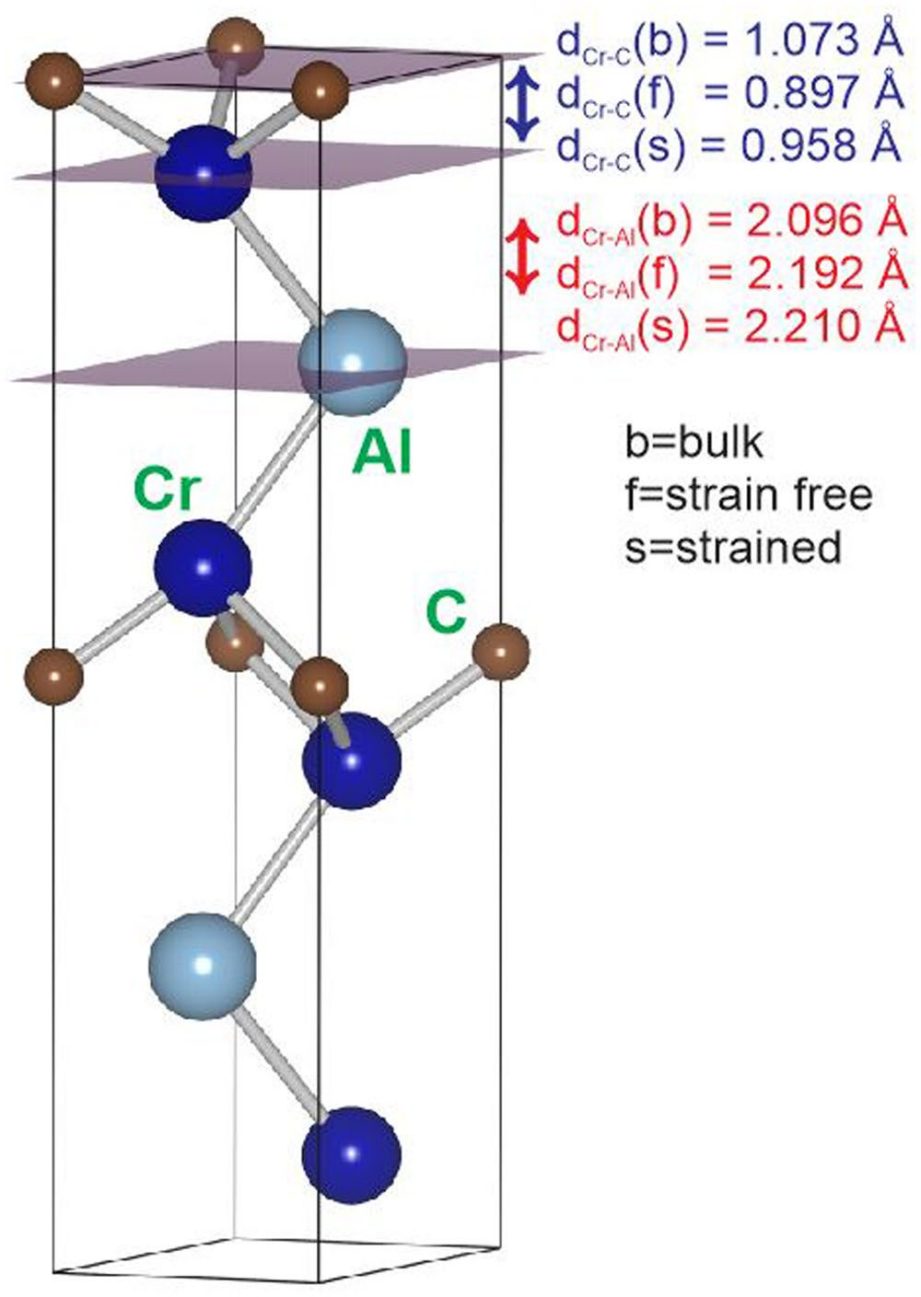
Unit cell of Cr_2_AlC with highlighted surface and two subsurface planes. Surface relaxations are described by the changes in the interplanar distances.

To correlate the electronic structure changes upon Cr2AlC surface formation and subsequent surface straining (Fig. 4) with the atomically resolved TEM data describing the local structure in the vicinity of pores (Figs 2 and 3), we analyse the surface relaxation effects at 0 K. Figure 5 shows the unit cell of Cr_2_AlC with the highlighted C-terminated (0001) surface and two subsurface planes. The Cr-C interplanar distance is decreased by 16% upon surface formation and then recovered to 89% of the pristine bulk interplanar distance. This is a consequence of the Cr d – C p bond weakening upon surface formation and its recovery upon straining, as discussed above (see Fig. 4). The Cr-Al interplanar distance exhibits completely a different behaviour. It is enlarged by 5% upon surface formation and subsequent straining, which is related to weakening of Cr d – Al p hybridized states (see Fig. 4). These observations are consistent with the shrinkage of Cr-C-Cr distance and elongation of Cr-Al interplanar distance observed experientially.

Investigations of three dimensional defects at high magnifications are scarce in literature. Xie *et al*.^37^ reported an *in-situ* observation of nanoscale gas bubbles at aluminium metal/oxide interface under the exposure of hydrogen in order to explain the commonly observed interfacial failure. Pruymboom *et al*.^38^ investigated 1–3 nm argon bubbles in sputtered Nb3Ge. The influence of the substrate bias potential on bubble formation in the thin film was studied and a threshold voltage between 100 and 150 V was identified for the bubble formation during the deposition. Hultman *et al*.^39^ reported the existence of nm-size voids at both column boundaries and within individual columns in titanium nitride thin films with columnar morphology. The formation mechanism was argued to be the trapping of nitrogen gas bubbles in epitaxial TiN films bounded by (002) planes. Music *et al*.^40^ studied 10 nm pores in amorphous boron suboxide thin films, suggesting that the noble gas atoms at high pressure can push out atoms of the amorphous material into the surroundings during the growth. Even though the origin of the observed voids is unclear, the presence thereof has large implications for physical and chemical properties. It is reasonable to assume that the presence of nanometre-scale defects will affect among others dislocation motion, crack formation, and transport properties. The nanometre-size pores have previously been overlooked in the literature on MAX phases. The occurrence of these nanometre-size defects is of significance for all physical and chemical properties. While many properties may be deteriorated upon pore formation, such as mechanical properties, implying that these pores should be avoided in practical applications, there are some transport properties that may benefit from their existence. For instance, to design efficient thermoelectrics, besides large Seebeck coefficient, low thermal conductivity and high electrical conductivity are required, but the electrical and thermal conductivity are interrelated as charge carriers contribute to the both phenomena^41^. Besides charge carriers, phonons also contribute to the thermal conductivity^41^. Forming pores, the phonon contribution to the overall thermal conductivity may significantly be reduced. This has been utilised in Si^42^ and may enable the design of MAX phases refractory materials regarding their thermal conductivity.

Furthermore, the existence of defects may affect the self-healing behaviour as well. Among many of the material systems with self-healing ability, including polymers, elastomers, fibre-reinforced polymer composites, cements, ceramics and metals, self-healing of ceramics has recently attracted much attention since the advantage of crack healing can immediately be combined with well-accepted, cutting-edge hard-coating technology and cost saving for the large-scale applications of critical mechanical parts, such as healing the cracks on the aircraft turbine^43^. MAX phases are one of the most promising ceramic materials for the self-healing applications.

## Conclusion

3D defects on the nanometre-scale near the phase-transformation region of HPPMS-deposited Cr_2_AlC thin films have been investigated by probe-corrected STEM. Severe lattice distortions have been observed for the first time on an atomic scale in the vicinity of these defects, where the Cr-Cr distance is shortened, and the Cr-Al distance is elongated compared to the strain-free regions in the specimen. Ab initio calculations, assuming that the observed defects are nm scale voids, have been performed to describe the effect of strain on the electronic structure of a C-terminated Cr_2_AlC(0001) surface. The formation of a Cr_2_AlC(0001) surface causes bond weakening of Cr d – C p as well as Cr d – Al p bonds compared to the bulk. While the Cr-C bonds recover their bulk strength when Cr_2_AlC(0001) is strained, the Cr-Al bonds experience only a partial recovery, still being weaker than their bulk counterparts. Hence, the relative strength difference between the Cr-C and Cr-Al bonds becomes larger when comparing bulk Cr_2_AlC and strained Cr_2_AlC(0001). This is consistent with the increase of the Cr-Al distance detected by STEM in the vicinity of nm-scale defects in Cr_2_AlC(0001) thin films.

## Experiment

The Cr_2_AlC film was deposited by high power pulse magnetron sputtering (HPPMS) in an industrial chamber (CC800/9, CemeCon AG). A compound target (50 × 8.8 × 1 cm^3^) produced via a powder metallurgical route consisting of Cr_3_C_2_, Cr and Al with a Cr:Al:C composition of 2:1:1 (supplied by Plansee Composite Materials GmbH) was used. A polycrystalline α-Al2O3 wafer (KERAFOL Keral 99, 1.5 × 1.5 × 0.038 cm^3^) was employed as substrate and heated to 600 °C prior to deposition. The sample to target distance was 5 cm. The set voltage, pulse on time and frequency were −1000 V, 50 μs and 333 Hz, respectively. The peak power density was 286 W/cm^2^ and the time averaged power was 984 W. The base pressure was below 0.55 mPa and the Ar pressure during deposition was 390 mPa. The deposition was performed at a substrate temperature of 600 °C and at floating potential for 180 min.

The TEM sample was prepared with the focused ion beam (FIB) in a dual-beam FEI Helios 660 microscope and by employing a standard lift-out procedure. The sample was first protected by a 100 nm Pt-layer deposited with a 5 kV electron beam, followed by a 3 μm Pt-layer deposited with the ion beam. The FIB ion energy was 30 kV. After the lift-out, an Omniprobe 5-post copper grid was used to host the TEM lamella, which was then transferred into an ion milling system (Fischione Nano-Mill) for post ion thinning with a low energy Ar ion-beam (500 eV) in an attempt to remove FIB damaged surface layers. The lamella was tilted to +10 and −10 degrees for 10 minutes each for the Nano-Mill process to minimize damage caused by low energy-ions.

## Electronic supplementary material

Supplementary Information

## References

[1] Barsoum MW, ElRaghy T (1996). Synthesis and characterization of a remarkable ceramic: Ti_3_SiC_2_. J. Am. Ceram. Soc..

[2] Eklund P, Beckers M, Jansson U, Hogberg H, Hultman L (2010). The M_n+1_AX_n_ phases: Materials science and thin-film processing. Thin Solid Films.

[3] Sun ZM (2011). Progress in research and development on MAX phases: a family of layered ternary compounds. Int. Mater. Rev..

[4] Barsoum MW, Radovic M (2011). Elastic and Mechanical Properties of the MAX Phases. Annu. Rev. Mater. Res..

[5] Sun ZM, Zhou YC, Li MS (2001). Oxidation behaviour of Ti_3_SiC_2_-based ceramic at 900–1300 degrees C in air. Corros. Sci..

[6] Emmerlich J (2007). Thermal stability of Ti_3_SiC_2_ thin films. Acta Mater..

[7] Barsoum MW (2000). The M(N+1)AX(N) phases: A new class of solids; Thermodynamically stable nanolaminates. Prog. Solid State Chem..

[8] To Baben M, Shang L, Emmerlich J, Schneider JM (2012). Oxygen incorporation in M_2_AlC (M = Ti, V, Cr). Acta Mater..

[9] Berger O, Boucher R, Ruhnow M (2015). Part I. Mechanism of oxidation of Cr_2_AlC films in temperature range 700–1200 degrees C. Surf. Eng..

[10] Music D, Sun Z, Ahuja R, Schneider JM (2007). Surface energy of M_2_AC(0001) determined by density functional theory (M = Ti, V, Cr; A = Al, Ga, Ge). Surf. Sci.

[11] Sun ZM, Ahuja R (2006). Ab initio study of the Cr_2_AlC (0001) surface. Appl. Phys. Lett..

[12] Sun ZM, Zhou J, Music D, Ahuja R, Schneider JM (2006). Phase stability of Ti_3_SiC_2_ at elevated temperatures. Scr. Mater.

[13] Cover MF, Warschkow O, Bilek MMM, McKenzie DR (2009). A comprehensive survey of M2AX phase elastic properties. J. Phys.: Condens. Matter.

[14] Music D, Schneider JM (2007). The correlation between the electronic structure and elastic properties of nanolaminates. JOM.

[15] Ingason AS (2014). A Nanolaminated Magnetic Phase: Mn_2_GaC. Mater. Res. Lett..

[16] Liao T, Wang JY, Zhou YC (2008). First-principles investigation of intrinsic defects and (N, O) impurity atom stimulated Al vacancy in Ti_2_AlC. Appl. Phys. Lett..

[17] Rosen J (2008). Oxygen incorporation in Ti_2_AlC thin films. Appl. Phys. Lett..

[18] Emmerlich J (2004). Growth of Ti3SiC2 thin films by elemental target magnetron sputtering. J. Appl. Phys..

[19] Eklund P (2007). Homoepitaxial growth of Ti–Si–C MAX-phase thin films on bulk Ti_3_SiC_2_ substrates. J. Cryst. Growth.

[20] Sun Z, Ahuja R, Li S, Schneider JM (2003). Structure and bulk modulus of M_2_AlC (M = Ti, V, and Cr). Appl. Phys. Lett..

[21] Hajas DE (2011). Oxidation of Cr_2_AlC coatings in the temperature range of 1230 to 1410 C. Surf. Coat. Technol..

[22] Heggen M, Luysberg M, Tillmann K (2016). FEI Titan 80–300 STEM. Journal of Large-scale Research Facilities.

[23] Barthel, J. D. Probe – STEM multislice image calculation program; Ernst Ruska-Centre for Microscopy and Spectroscopy with Electrons: Jülich, Germany; www.er-c.org.

[24] Rosen J (2006). Reducing the impurity incorporation from residual gas by ion bombardment during high vacuum magnetron sputtering. Appl. Phys. Lett..

[25] Ying G (2011). Effect of Cr_7_C_3_ on the mechanical, thermal, and electrical properties of Cr_2_AlC. J. Alloys Compd..

[26] Huntz AM, Maréchal L, Lesage B, Molins R (2006). Thermal expansion coefficient of alumina films developed by oxidation of a FeCrAl alloy determined by a deflection technique. Appl. Surf. Sci..

[27] Petrov I, Barna PB, Hultman L (2003). Microstructural evolution during film growth. J. Vac. Sci. Technol., A.

[28] Schneider JM, Rohde S, Sproul WD, Matthews A (2000). Recent developments in plasma assisted physical vapour deposition. J. Phys. D: Appl. Phys.

[29] Thornton JA, Hoffman DW (1989). Stress-related effects in thin films. Thin Solid Films.

[30] Hohenberg P, Kohn W (1964). Inhomogeneous electron gas. Phys. Rev. B.

[31] Kresse G, Hafner J (1993). Ab-initio molecular dynamics for open shell transition metals. Phys. Rev. B.

[32] Perdew JP, Burke K, Ernzerhof M (1996). Generalized gradient approximation made simple. Phys. Rev. Lett..

[33] Monkhorst HJ, Pack JD (1976). Special points for Brillouin-zone integrations. Phys. Rev. B.

[34] Blochl PE (1994). Projector augmented-wave method. Phys. Rev. B.

[35] Lin Z, Zhou Y, Li M (2007). Synthesis, microstructure, and properties of Cr_2_AlC. J. Mater. Sci. Technol..

[36] Emmerlich J, Music D, Houben A, Dronskowski R, Schneider JM (2007). Systematic study on the pressure dependence of M_2_AlC phases (M = Ti,V,Cr,Zr,Nb,Mo,Hf,Ta,W). Phys. Rev. B.

[37] Xie D (2015). *In situ* study of the initiation of hydrogen bubbles at the aluminium metal/oxide interface. Nat. Mater..

[38] Pruymboom A, Berghuis P, Kes PH (1987). Threshold for argon bubble growth in sputtered amorphous Nb_3_Ge. Appl. Phys. Lett..

[39] Hultman L, Sundgren J-E, Greene JE, Bergstrom DB, Petrov I (1995). High‐flux low‐energy (≂20 eV) N^+2^ ion irradiation during TiN deposition by reactive magnetron sputtering: Effects on microstructure and preferred orientation. J. Appl. Phys..

[40] Music D, Kreissig U, Czigany Z, Helmersson U, Schneider JM (2003). Elastic modulus-density relationship for amorphous boron suboxide thin films. Appl. Phys. A.

[41] Snyder G, Toberer E (2008). Complex thermoelectric materials. Nat. Mater..

[42] Alvarez FX, Jou D, Sellitto A (2010). Pore-size dependence of the thermal conductivity of porous silicon: A phonon hydrodynamic approach. Appl. Phys. Lett..

[43] Sloof, W. G. Self healing in coatings as high temperatures in *Self healing materials* (ed. van der Zwaag, S.) 309–321 (Springer, 2007).

