# Supplementary material for: Nanometre-scale 3D defects in Cr2AlC thin films
Source: Sci Rep. 2017 Apr 20;7:984. doi: 10.1038/s41598-017-01196-3 (PMC5430507; doi:10.1038/s41598-017-01196-3)
Supplement: Supplementary file 1 — Supplementary Information [file 41598_2017_1196_MOESM1_ESM.pdf]

Supplementary Information : Nanometre-scale 3D defects in Cr2AlC thin films

Y. T. Chen[1,*], D. Music[1] , L. Shang[1] , J. Mayer[2,3], J. M. Schneider[1]

[1]Materials Chemistry, RWTH Aachen University, Kopernikusstr. 10, 52074 Aachen, Germany

[2]Central Facility for Electron Microscopy, RWTH Aachen University, 52056 Aachen, Germany

[3]Ernst Ruska-Centre for Microscopy and Spectroscopy with Electrons, Forschungszentrum Juelich, 52425 Juelich, Germany

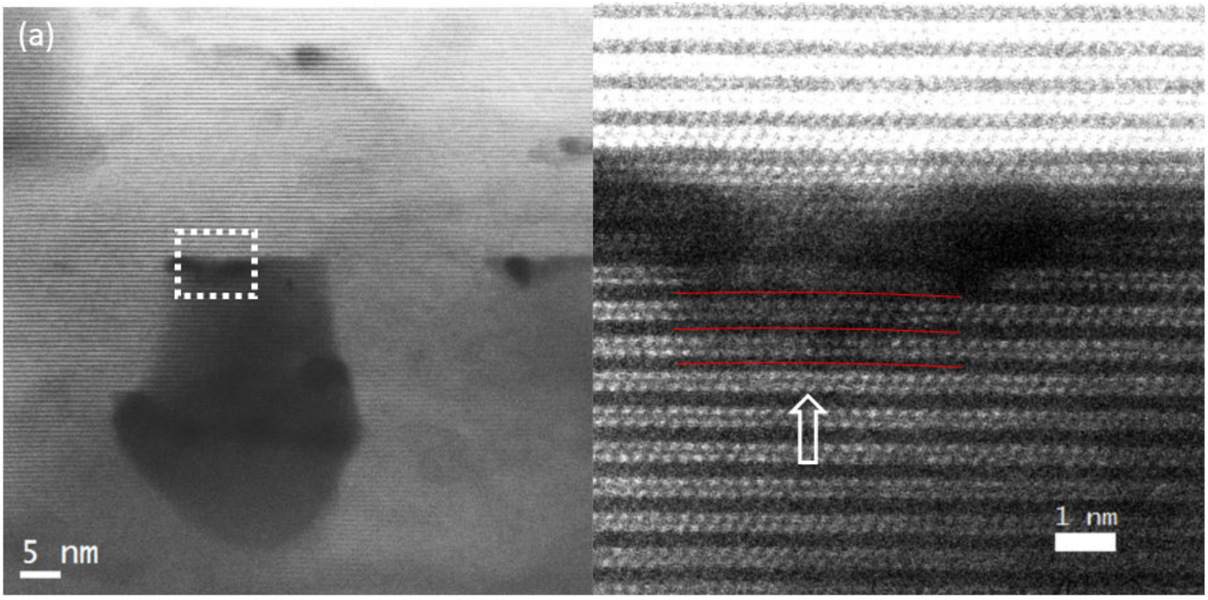

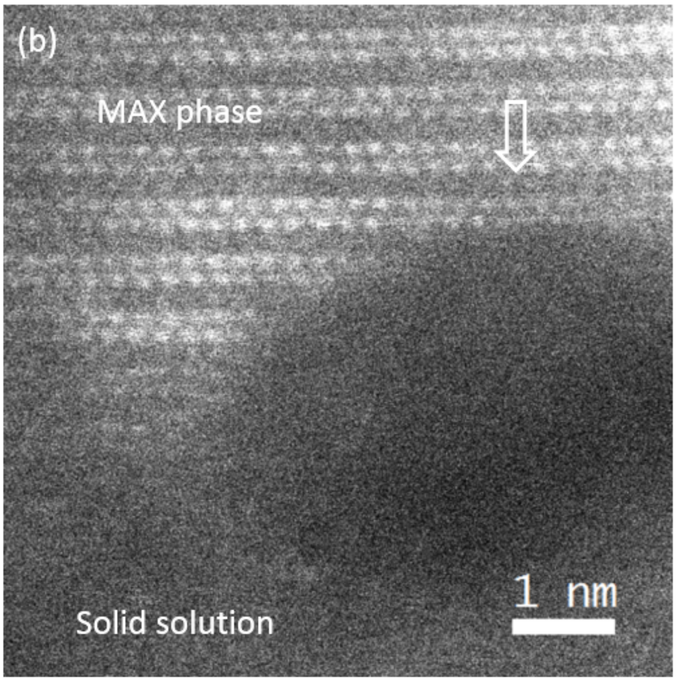

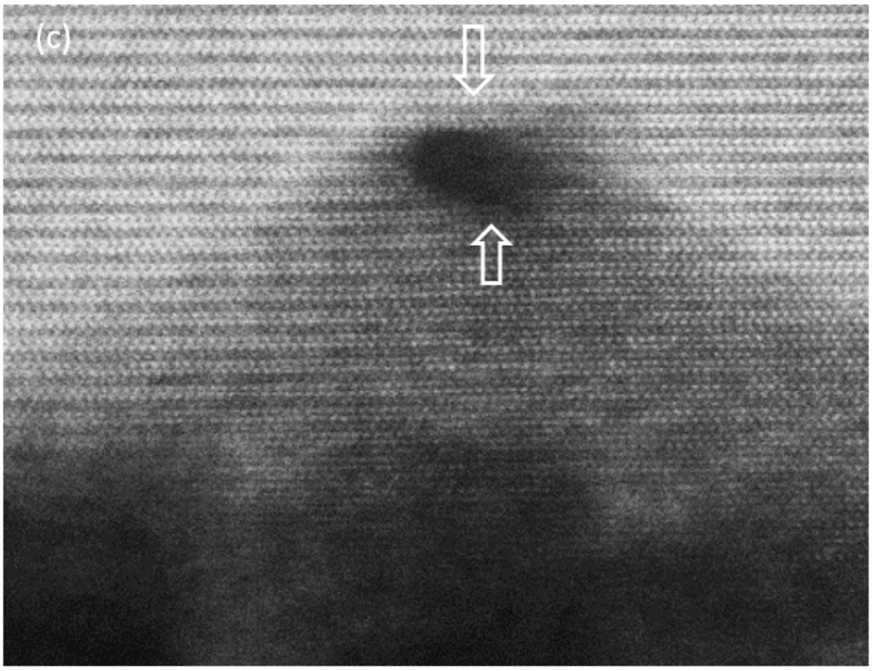

Figure S1. (a-c) Examples of lattice distortions in the vicinity of the defects on the same sample.
